# Diminished osteoprotegerin production by gingival fibroblasts from periodontitis patients is associated with their reduced ability to suppress osteoclastogenesis

**DOI:** 10.1038/s41598-025-29944-w

**Published:** 2025-11-28

**Authors:** Elwira Nieboga, Kamila Drzazga, Alicja Plonczynska, Dominika M. Drapala, Aureliusz Schuster, Mariia Melnykova, Julia Skawska, Michal Zajdel, Malgorzata Kantorowicz, Sanne Roffel, Joanna Cichy, Marta Czesnikiewicz-Guzik, Tomasz Kaczmarzyk, Susan Gibbs, Jan Potempa, Aleksander M. Grabiec

**Affiliations:** 1 Department of Microbiology, Faculty of Biochemistry, Biophysics and Biotechnology, Jagiellonian University, Kraków, Poland; 2 Doctoral School of Exact and Natural Sciences, Jagiellonian University, Kraków, Poland; 3 Department of Immunology, Faculty of Biochemistry, Biophysics and Biotechnology, Jagiellonian University, Kraków, Poland; 4 Chair of Oral Surgery, Faculty of Medicine, Jagiellonian University Medical College, Kraków, Poland; 5 Department of Periodontology, Preventive Dentistry and Oral Medicine, Faculty of Medicine, Jagiellonian University Medical College, Kraków, Poland; 6 Department of Oral Cell Biology, Academic Centre for Dentistry Amsterdam (ACTA), University of Amsterdam and Vrije Universiteit Amsterdam, Amsterdam, The Netherlands; 7 Oral Sciences Research Group, School of Medicine, Dentistry & Nursing, College of Medical, Veterinary and Life Sciences, University of Glasgow, Glasgow, UK; 8 Department of Molecular Cell Biology and Immunology, Amsterdam Infection and Immunity Institute, Amsterdam University Medical Centre Location Vrije Universiteit Amsterdam, Amsterdam, The Netherlands; 9 Department of Oral Immunology and Infectious Diseases, University of Louisville School of Dentistry, Louisville, KY USA

**Keywords:** Cell biology, Diseases, Immunology, Medical research

## Abstract

Osteoclast-mediated alveolar bone resorption is a key hallmark of periodontitis. Gingival fibroblasts (GFs) play multifaceted roles in the progression of chronic inflammation; however, their involvement in osteoclastogenesis remains controversial. Here, we analyzed the effects of mediators secreted by GFs from healthy donors and periodontitis patients on osteoclast formation. We observed strong suppression of receptor activator of nuclear factor kappa-B ligand (RANKL)-induced osteoclastogenesis by conditioned media from healthy donor GFs, which was independent of any prior in vitro stimulation or infection. These media contained high levels of osteoprotegerin (OPG), and GFs were identified as the main OPG source in gingival tissue using a 3D organotypic reconstructed human gingiva model and a single-cell RNA-seq dataset. Notably, GFs from periodontitis patients produced significantly less OPG compared to GFs from healthy individuals and were less effective at suppressing osteoclastogenesis. Finally, siRNA-mediated silencing of OPG expression in GFs from periodontitis patients further decreased their inhibitory effect on osteoclast formation, confirming the central role of OPG in osteoclastogenesis regulation by GFs. Collectively, these results demonstrate that in healthy gingival tissue, GFs exert bone-protective effects by inhibiting osteoclast formation. However, prolonged exposure to the microenvironment of the inflamed gingival tissue could impair this protective function through sustained reduction of OPG production, which may contribute to alveolar bone resorption in periodontitis.

**Supplementary Information:**

The online version contains supplementary material available at 10.1038/s41598-025-29944-w.

## Introduction

Periodontitis is a chronic inflammatory disease of the periodontium that affects approximately 11% of the global population in its advanced stages^1^. It not only causes tooth loss but is also associated with numerous systemic diseases^2^. While the disease is initiated by microbial imbalance, it is the host’s excessive immune response that drives chronic inflammation^3^. Immunopathological processes in the periodontium, characterized by immune cell infiltration and the accumulation of inflammatory mediators, ultimately lead to osteoclast-mediated alveolar bone resorption, a hallmark of periodontitis progression and the main clinical challenge in its treatment^4^.

Osteoclasts are tartrate-resistant acid phosphatase (TRAP)-positive cells containing more than three nuclei. Within the family of multinucleated giant cells (MNGCs), osteoclasts are the only cell type capable of resorbing bone^5^. During osteoclastogenesis, monocytes fuse in response to various cytokines, leading to their differentiation into mature osteoclasts^6^. One of the key cytokines regulating osteoclastogenesis is receptor activator of nuclear factor-κB ligand (RANKL), which binds to RANK on the surface of osteoclast precursors. Osteoprotegerin (OPG), encoded by the *TNFRSF11B* gene, functions as a decoy receptor that binds RANKL, thereby preventing its interaction with RANK^7^. The ratio of OPG to RANKL is considered a critical determinant of bone resorption activity^8^. Indeed, RANKL levels are elevated, whereas OPG levels are diminished in patients with periodontitis^8,9^, promoting osteoclast formation, which contributes to alveolar bone degradation and, ultimately, tooth loss. Notably, conventional periodontal treatment lowers the RANKL/OPG ratio at sites of active disease, leading to a reduction in osteoclast numbers and, consequently, decreased bone resorption^10^. In inflamed gingival tissue, infiltrating immune cells, particularly activated T and B lymphocytes, are the primary source of RANKL^11^. Additionally, one of the key periodontopathogens, *Porphyromonas gingivalis*, has also been shown to enhance RANKL production in osteoblasts^12^. Lam et al. also demonstrated that tumor necrosis factor (TNF) can promote osteoclastogenesis, reinforcing the idea that inflammatory conditions favor bone resorption^13^.

Gingival fibroblasts (GFs) are among the most abundant cell types in gingival connective tissue^14^. While their primary role is to maintain tissue structure and integrity, numerous studies have highlighted their important role in the progression of periodontal inflammation^14^. GFs modulate immunity primarily through their effects on immune cells infiltrating gingival tissue, and a recent single-cell transcriptomic study revealed the central role of the interplay between GFs and neutrophils in the pathogenesis of periodontitis^15^. Consistent with this, we previously demonstrated that GFs can promote neutrophil recruitment to gingival tissue through the synergistic induction of prostaglandin E2 (PGE2) production in response to simultaneous exposure to oral pathogens and inflammatory cytokines, which subsequently enhances *IL8* expression in macrophages^16^. GFs also produce a wide range of mediators that directly or indirectly regulate osteoclastogenesis, although their role in alveolar bone homeostasis remains controversial. While in vitro evidence suggests that GFs predominantly produce OPG and have a bone-protective role under homeostatic conditions^17^, studies of the roles of GFs in osteoclastogenesis under inflammatory conditions have produced conflicting results^18,19^. Importantly, analyses directly comparing the ability of GFs from healthy donors and periodontitis patients to regulate osteoclastogenesis, as well as the factors involved in this process, are lacking. Therefore, in this study, we analyzed the effects of mediators secreted by GFs subjected to inflammatory stimulation or bacterial infection on RANKL-induced osteoclastogenesis. Next, we used both 2D GF monocultures and a 3D organotypic reconstructed human gingiva (RHG) model, as well as a single-cell RNA-seq dataset, to verify the contribution of GFs to OPG production in healthy gingiva. Finally, we compared the ability of GFs from healthy donors and periodontitis patients to influence osteoclast differentiation, and conducted a detailed analysis of GF-derived OPG’s role in this process.

## Results

### GF-derived conditioned media suppress RANKL-mediated osteoclastogenesis

**Fig. 1:**
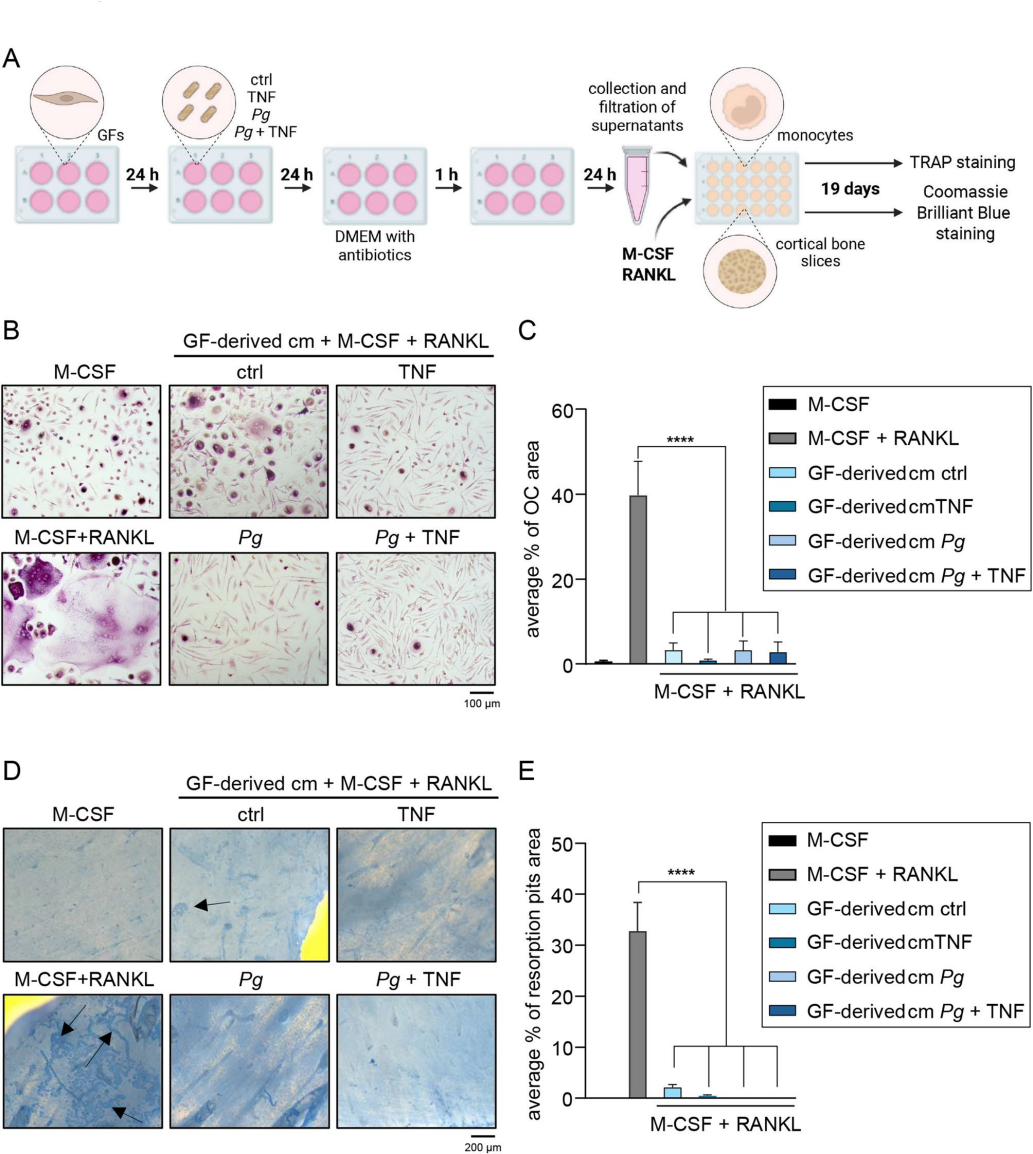
GF-derived conditioned media suppress osteoclastogenesis. (**A**) Schematic representation of the experimental design. Created in BioRender. Grabiec, A. (2025) https://BioRender.com/j951hwr. Monocytes were cultured for 19 days on plastic (**B**,**C**) or bone (**D**,**E**) in the presence of M-CSF, with or without RANKL (10 ng/ml). Culture media consisted of αMEM supplemented with 10% FBS, used either alone or mixed 1:1 with conditioned medium (cm) collected from healthy donor GFs (*n* = 6) that were left untreated (ctrl), infected with *P. gingivalis* (*Pg*, MOI = 20), stimulated with TNF (10 ng/ml) or treated with both factors. Cm from individual donors were pooled prior to use. (**B**) Representative images of TRAP-stained cells from three independent experiments using monocytes from different donors treated with one pool of conditioned media. (**C**) Quantification of the average percentage of surface area covered by osteoclasts (*n* = 3). (**D**) Representative images of stained bone slices from two independent experiments using monocytes from different donors. Black arrows indicate resorption pits. (**E**) Quantification of the average percentage of surface area of resorption pits (*n* = 5; where “n” refers to separate microscopic fields). Data are presented as the mean + SEM. ****P < 0.0001; One-way ANOVA followed by Bonferroni multiple comparison test.

To address the controversies surrounding the role of GFs in regulating osteoclastogenesis in periodontal disease, we collected conditioned media from healthy donor GFs that were either left untreated or infected with *P. gingivalis*, with or without TNF stimulation, to mimic the inflammatory environment of periodontitis-affected gingival tissue. These media, supplemented with M-CSF and RANKL, were then added to monocyte cultures that were maintained for 19 days and subsequently stained for TRAP activity (Fig. 1A). Cells cultured with M-CSF or M-CSF and RANKL without conditioned media were used as negative and positive controls, respectively. As expected, cells treated with M-CSF alone did not fuse into osteoclasts. In contrast, treatment with M-CSF and RANKL induced extensive osteoclast fusion, with multinucleated cells covering approximately 40% of the image area. Notably, few osteoclasts were observed in cultures treated with M-CSF and RANKL in the presence of GF-derived conditioned media, regardless of GF treatment conditions (Fig. 1B-C). These results demonstrate that GFs, regardless of their inflammatory status, secrete mediators that effectively suppress osteoclastogenesis.

To confirm that osteoclasts generated using this protocol are functional, we seeded monocytes onto bovine cortical bone slices and treated them with GF-derived conditioned media in the presence of M-CSF and RANKL for 19 days. Bone slices were subsequently stained to visualize resorption pits, which reflect osteoclast bone-resorptive activity (Fig. 1D). Multiple resorption pits appeared on slices treated only with M-CSF and RANKL. A limited resorption area was also detected on slices treated with conditioned media from unstimulated GFs. In contrast, no resorption pits were observed on bone slices treated with conditioned media derived from GFs that were infected or stimulated, indicating the absence of active osteoclasts (Fig. 1E). These findings confirm that soluble factors secreted by GFs from healthy individuals suppress RANKL-mediated osteoclastogenesis and suggest that this effect is not affected by acute inflammatory cell activation in vitro.

**Fig. 2:**
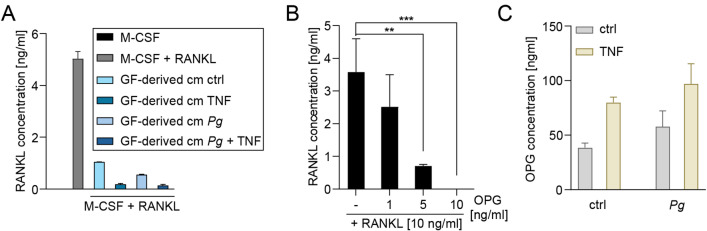
GF-derived OPG neutralizes RANKL. (**A**) RANKL concentration in osteoclast culture supernatants after 19-day culture with M-CSF, RANKL (both at 10 ng/ml), and conditioned media (cm) derived from healthy donor GFs prepared as in Fig. 1. Media with stimulants were refreshed 24 h before collecting supernatants. (**B**) Detection of RANKL in solutions containing recombinant human RANKL (10 ng/ml) and increasing concentrations of recombinant human OPG (1, 5, 10 ng/ml). (**C**) OPG concentration in pooled conditioned media derived from GFs from 6 healthy donors prepared as in Fig. 1. Data are presented as (A, C) the mean of two measurements + SD or as (B) the mean of three measurements + SD. **P < 0.01; ***P < 0.001; One-way ANOVA followed by Bonferroni multiple comparison test.

Next, to identify the mechanism underlying the observed suppression of osteoclastogenesis, we measured the levels of RANKL in the supernatants from osteoclast cultures analyzed in the previous experiment. With the exception of the negative control containing M-CSF alone, all analyzed supernatants had been supplemented with 10 ng/ml RANKL prior to incubation with monocytes (Fig. 2A). The amounts of RANKL detected in the supernatants from cultures treated with M-CSF and RANKL were approximately 50% lower than the RANKL concentration added to the cell culture, possibly due to its consumption or binding to culture plates and FBS proteins. In contrast, a much more pronounced decrease in RANKL concentrations was observed in the supernatants from cultures treated with GF-derived conditioned media, particularly when GFs had been stimulated with TNF. Since GFs are known to produce high levels of OPG, a decoy receptor that binds to RANKL, we hypothesized that GF-derived OPG present in the conditioned media may be responsible for the observed reduction in RANKL levels. Indeed, incubation of RANKL (10 ng/ml) in vitro with increasing concentrations of recombinant OPG led to a dose-dependent reduction in detectable RANKL concentrations, with complete neutralization observed at an OPG concentration of 10 ng/ml (Fig. 2B), confirming effective binding of OPG to RANKL that prevents detection of the latter by ELISA. Finally, we measured the concentration of OPG in the conditioned media derived from healthy donor GFs and found it to be remarkably high, ranging from 50 to 100 ng/ml depending on the GF treatment condition with notable but modest trends towards increased OPG production following TNF stimulation or bacterial infection (Fig. 2C). These results suggest that the high levels of OPG present in GF-derived conditioned media, regardless of the presence or absence of inflammatory stimulation are likely responsible for the observed suppression of osteoclastogenesis.

### GFs are the primary source of OPG in RHG

**Fig. 3:**
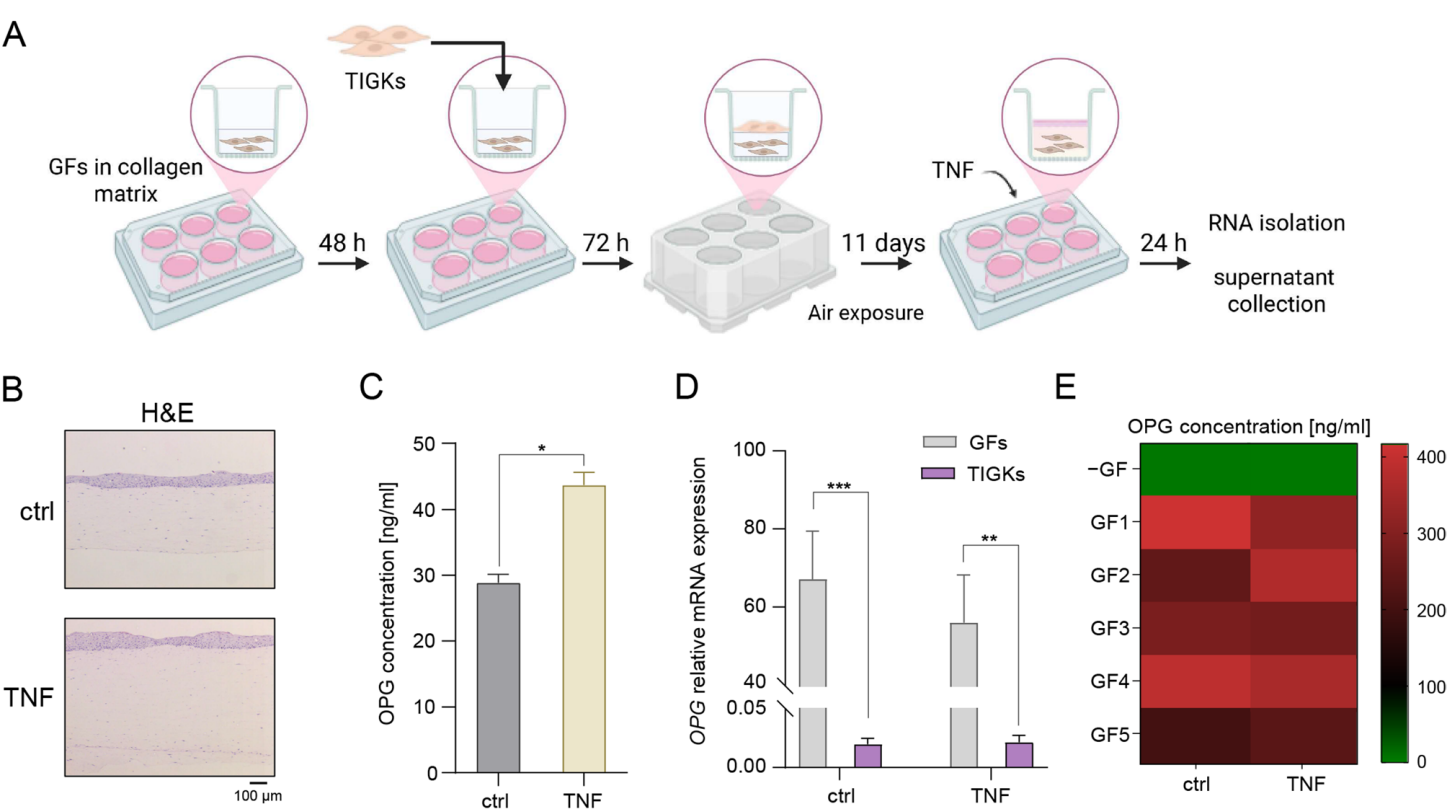
GFs are the primary source of OPG in RHG. (**A**) Schematic representation of the construction of the organotypic RHG model. Created in BioRender. Grabiec, A. (2025) https://BioRender.com/n2x08ku. (**B**) Representative images of RHG paraffin sections stained with hematoxylin and eosin (H&E). (**C**) OPG production by RHG that were left untreated (ctrl) or were stimulated with 1 ng/ml TNF for 24 h (*n* = 3). (**D**) qPCR analysis of *OPG* and *RANKL* expression in GF and TIGK fractions isolated from RHG (*n* = 5). (**E**) OPG production by unstimulated (ctrl) or TNF-stimulated (1 ng/ml) RHG constructed without GFs (− GF) or with GFs from five healthy donors (GF1-5) embedded in the collagen matrix. Data are presented as the mean + SEM (C, D) or as a heatmap (E). *P < 0.05; **P < 0.01; ***P < 0.001; paired t test (C); unpaired t test (D).

To confirm that the ability of GFs to produce large amounts of OPG is preserved in a more complex in vitro model that simulates human gingival tissue, we developed a 3D organotypic RHG model (Fig. 3A). The composition and structural integrity of the RHG was confirmed using hematoxylin and eosin staining (Fig. 3B). Similarly to GF monocultures, RHG produced high levels of OPG (approximately 30 ng/ml), which were further increased upon TNF stimulation. (Fig. 3C). To identify the cellular source of OPG within the RHG, we separated keratinocytes and GFs from the RHG after 24 h of stimulation with TNF and extracted RNA from each fraction for gene expression analysis. Relative mRNA expression of *OPG* was significantly higher in the GF fraction compared to the TIGK fraction (Fig. 3D). In contrast, *RANKL* expression was either undetectable or only detectable at very high threshold cycle values (data not shown), confirming that structural gingival cells are not a relevant source of RANKL in gingival tissue. To verify these findings at the protein level, we constructed RHG models with or without GFs from healthy donors in the collagen matrix. Consistent with the mRNA data, RANKL was undetectable in the supernatants from any of the analyzed RHGs (data not shown), whereas high concentrations of OPG were only produced by RHGs containing GFs (Fig. 3E).

**Fig. 4:**
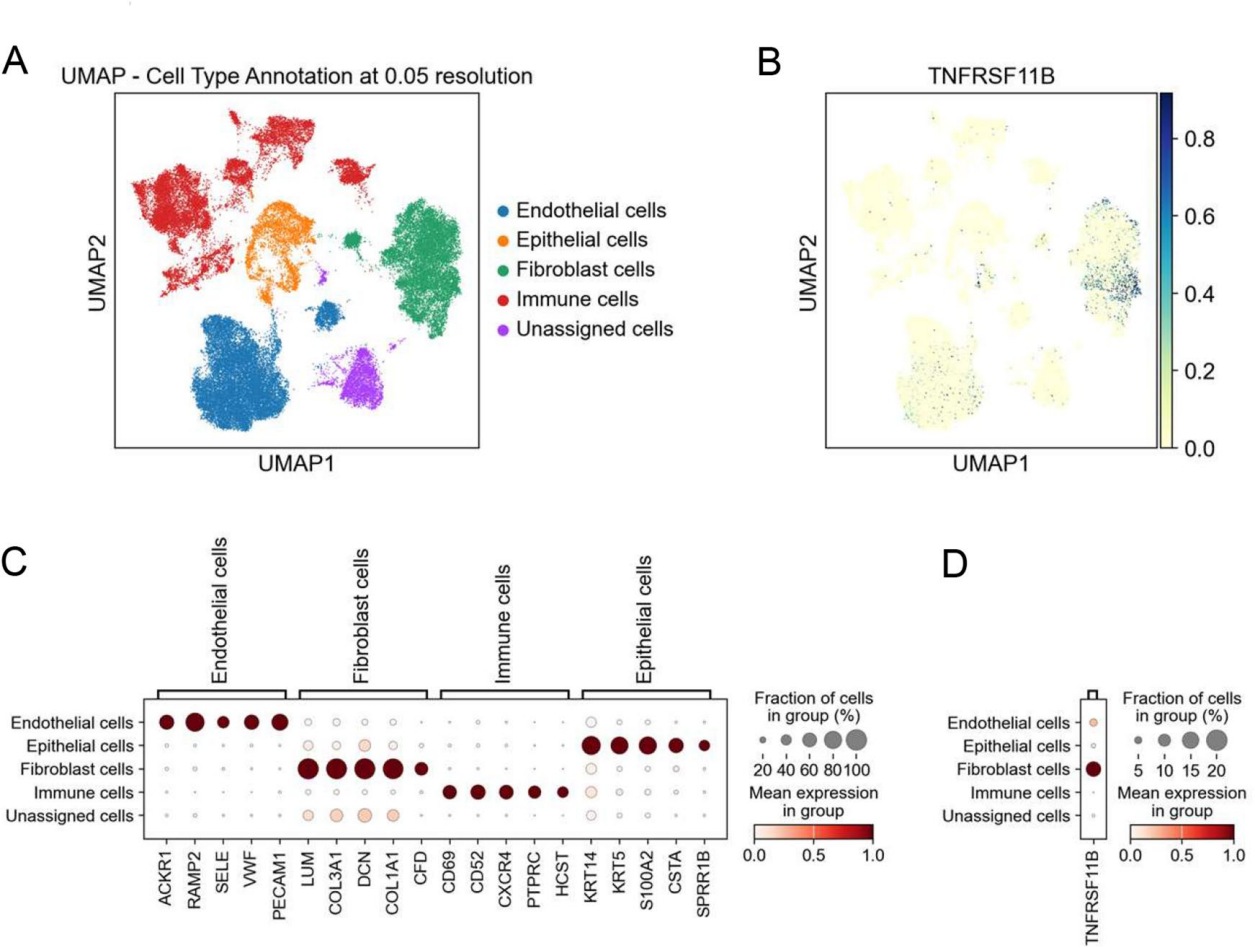
GFs are the main cell type expressing *OPG* in human healthy gingival tissue. Analysis of the single-cell RNA-seq data from healthy gingival tissue (*n* = 13) based on dataset GSE164241. UMAP representations of (**A**) major cell populations and (**B**) *TNFRSF11B* (*OPG*) expression. Dot plots illustrating (**C**) the expression of cell type-specific markers and (**D**) *TNFRSF11B* (*OPG*) expression across major cell populations. Expression values are normalized and scaled averages.

To independently verify these observations in healthy gingival tissue, we analyzed a single-cell RNA-seq dataset from the study of Williams et al.^15^, which described a human oral mucosa cell atlas. Using the well-stablished cell type-specific marker genes defined in the original study to distinguish four major cell types (Fig. 4A and C), we found that the *TNFRSF11B* gene, which encodes OPG, is expressed at the highest levels in cells classified as fibroblasts. In contrast, lower levels of *TNFRSF11B* expression were detected in endothelial cells, whereas its expression was negligible in immune and epithelial cells (Fig. 4B and D). These results confirm that GFs are the primary source of OPG in gingival tissue.

### GFs from periodontitis patients produce less OPG compared to GFs from healthy donors

**Fig. 5:**
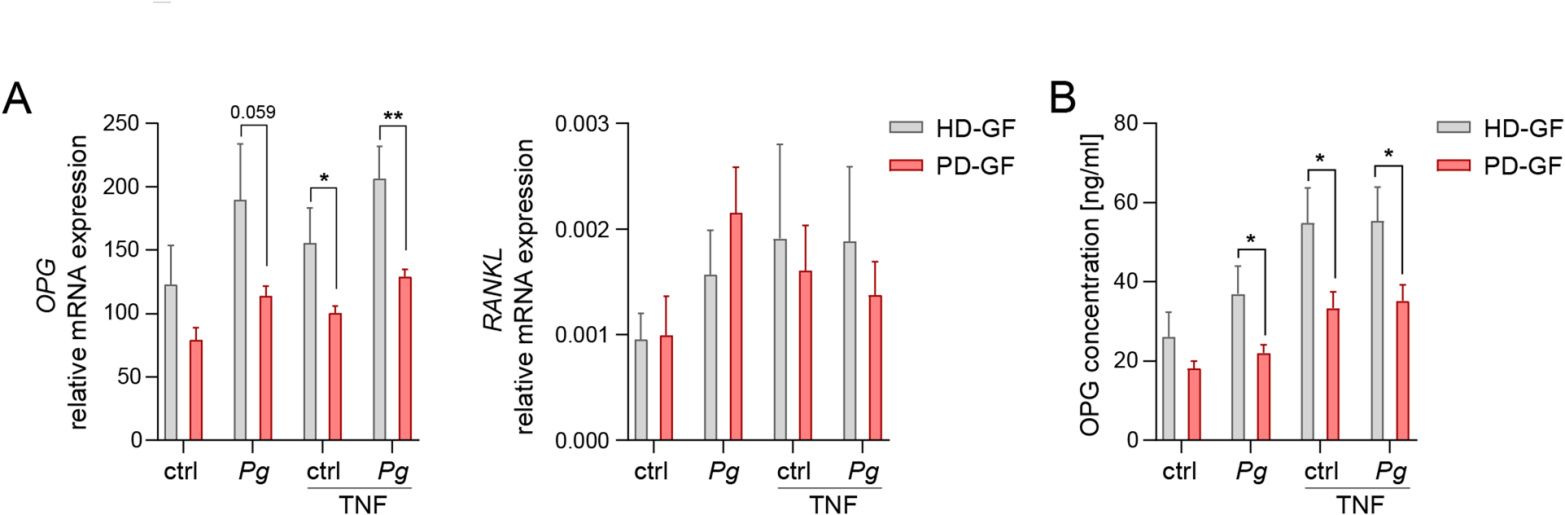
GFs from periodontitis patients produce lower levels of OPG than healthy donor GFs. GFs obtained from healthy donors (HD-GFs) or patients with periodontitis (PD-GFs) were left untreated (ctrl) or were infected with *P. gingivalis* (*Pg*) (MOI = 20), in the absence or presence of TNF (10 ng/ml), for 4 h (**A**) or 24 h (**B**). (A) qPCR analysis of *OPG* and *RANKL* expression (*n* = 7). (B) OPG protein levels in GF supernatants (*n* = 4). Data are presented as the mean + SEM. *P < 0.05; **P < 0.01; unpaired t test.

Given the conflicting data regarding the role of GFs in osteoclast formation and the clearly bone-protective activity of GF-secreted products observed in our experiments, we hypothesized that GFs from healthy donors and periodontitis patients may differ in their abilities to regulate osteoclastogenesis. To test this, we compared the expression of *OPG* and *RANKL* in GFs from healthy individuals and patients with periodontitis infected with *P. gingivalis* in the presence or absence of TNF (Fig. 5A left panel). Across all tested conditions, GFs from periodontitis patients consistently expressed lower mRNA levels of *OPG* than cells from healthy donors. In contrast, no differences were observed in *RANKL* expression, which remained very low in both groups (Fig. 5A right panel). Consistent with the mRNA data, GFs from periodontitis patients produced significantly less OPG protein than those from healthy donors under all experimental conditions (Fig. 5B), whereas RANKL protein was undetectable (data not shown). These observations suggest that prolonged exposure to the local inflammatory environment of gingival tissue may alter *OPG* expression in GFs, thereby modulating their ability to suppress osteoclastogenesis.

### Conditioned media from healthy donor GFs are more effective in suppressing osteoclastogenesis than media from periodontitis patient GFs

**Fig. 6:**
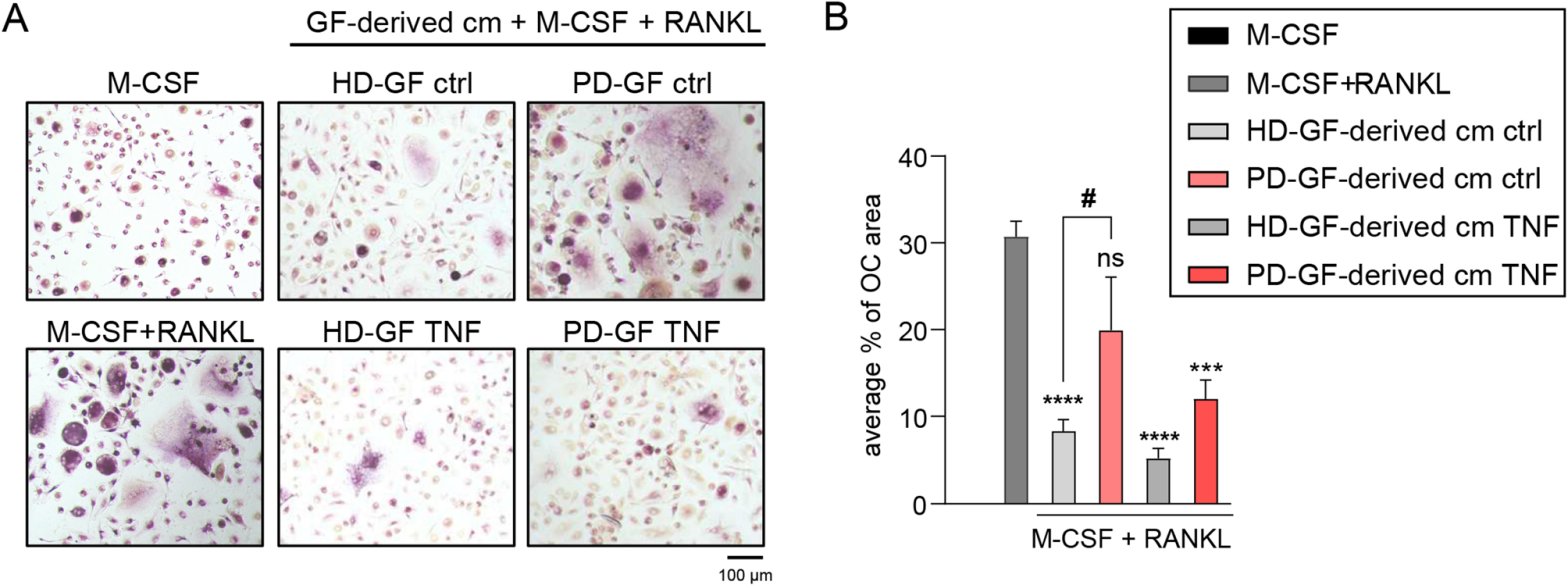
GFs from periodontitis patients have a diminished ability to suppress osteoclastogenesis compared to healthy donor GFs. Monocytes were cultured for 19 days in the presence of M-CSF (10 ng/ml), with or without RANKL (40 ng/ml). Culture media consisted of αMEM supplemented with 10% FBS, used either alone or mixed 1:1 with conditioned medium (cm) collected from GFs isolated from healthy donors (HD-GFs, *n* = 3) or patients with periodontitis (PD-GFs, *n* = 3) that were left untreated (ctrl) or were stimulated with TNF (10 ng/ml) and prepared for experiments as in Fig. 1. (**A**) Representative images of TRAP-stained cells from two independent experiments using monocytes from different donors treated with one pool of conditioned media. (**B**) Quantification of the average percentage of surface area occupied by osteoclasts (*n* = 5; where “n” refers to separate microscopic fields). Data are shown as the mean + SEM. ***P < 0.001; ****P < 0.0001; ns: not significant; One-way ANOVA followed by Bonferroni multiple comparison test performed relative to positive control (RANKL-treated cells without cm); #*P* < 0.05; unpaired t test.

To determine whether GFs from healthy donors and periodontitis patients differ in their ability to modulate osteoclastogenesis, we compared the effects of conditioned media from the two groups on RANKL-induced osteoclast formation. Conditioned media from unstimulated and TNF-stimulated GFs from three independent donors per group were pooled and their effects on RANKL-induced osteoclast differentiation were assessed (Fig. 1A). Due to the high concentrations of OPG in the conditioned media, a higher RANKL concentration (40 ng/ml) was used in this experiment. While conditioned media from unstimulated healthy donor GFs markedly suppressed osteoclastogenesis, media from unstimulated GFs from periodontitis patients had a weaker inhibitory effect. A similar trend was observed with conditioned media from TNF-stimulated GFs, though their ability to inhibit osteoclast formation was increased compared to that of media from unstimulated cells, likely resulting from TNF-induced upregulation of OPG (Fig. 6A-B). These findings suggest that changes in the ability of GFs from periodontitis patients to inhibit osteoclastogenesis may partly explain the conflicting reports in the literature regarding the role of GFs in osteoclastogenesis.

### OPG produced by GFs is responsible for suppression of osteoclastogenesis

**Fig. 7:**
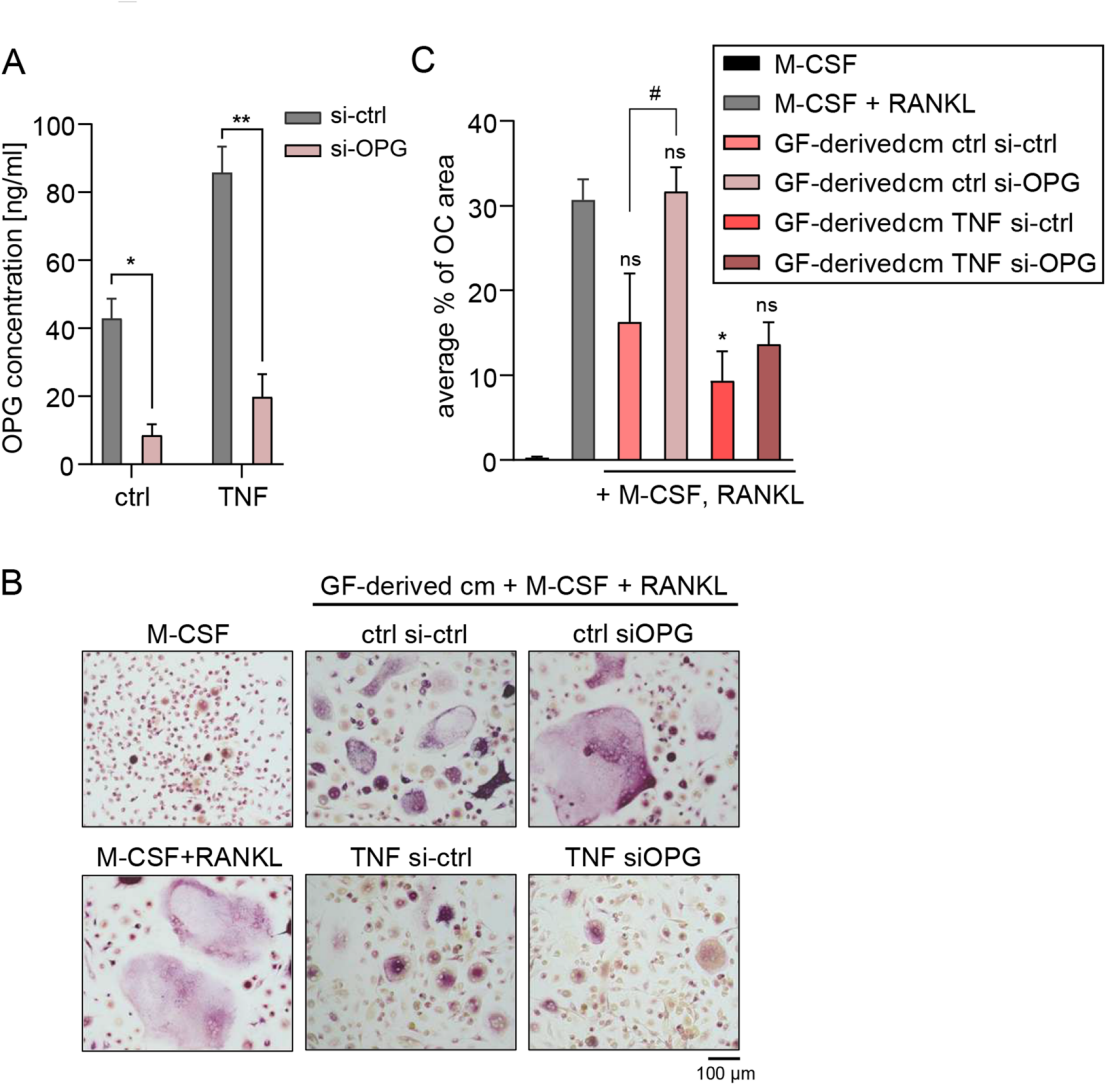
OPG produced by GFs is responsible for suppression of osteoclastogenesis by GF-derived conditioned media. (**A**) OPG production by GFs from periodontitis patients (*n* = 3) transfected with control non-targeting siRNA (si-ctrl) or *OPG*-targeting siRNA (si-OPG) before stimulation with TNF for 24 h. (**B**,**C**) Monocytes were cultured for 19 days in the presence of M-CSF (10 ng/ml), with or without RANKL (40 ng/ml). Culture media consisted of αMEM supplemented with 10% FBS, used either alone or mixed 1:1 with conditioned medium (cm) collected from GFs isolated from patients with periodontitis (PD-GFs, *n* = 3) that were transfected with si-ctrl or si-*OPG* followed by culture in the absence (ctrl) or presence of 10 ng/ml TNF for 24 h and downstream processing as in Fig. 1. (B) Representative images of TRAP-stained cells from three independent experiments using monocytes from different donors treated with one pool of conditioned media. (**C**) Quantification of the average percentage of surface area occupied by osteoclasts (*n* = 3). Data are shown as the mean + SEM. *P < 0.05; **P < 0.01; ns: not significant; paired t test (**A**); One-way ANOVA followed by Bonferroni multiple comparison test performed relative to positive control (RANKL-treated cells without cm) (C); #*P* < 0.05; unpaired t test (**C**).

Finally, we sought to determine whether OPG produced by GF is a principal factor responsible for the observed suppression of osteoclastogenesis by GF-derived conditioned media. To this end, we generated conditioned media from GFs with silenced OPG expression. Knockdown efficiency was confirmed by measuring OPG concentrations in GF supernatants (Fig. 7A). Conditioned media from GFs from three periodontitis patients treated with either control or OPG-specific siRNA were pooled and tested for their ability to suppress RANKL-induced osteoclastogenesis. Cultures treated with conditioned media from control siRNA-transfected GFs exhibited significantly reduced osteoclast area compared to those treated with media from GFs with silenced OPG expression. The latter showed osteoclast areas comparable to those of RANKL-treated controls, indicating that OPG contributes substantially to the inhibitory effect (Fig. 7B,C). When conditioned media from TNF-stimulated GFs were used, suppression of osteoclastogenesis was even more pronounced, consistent with previous experiments. However, the impact of OPG silencing was weaker compared to that induced by media from unstimulated GFs (Fig. 6B,C), possibly due to the higher levels of OPG in these samples (Fig. 7A). These findings confirm that OPG produced by GFs may play a key role in mediating the suppression of osteoclast formation.

## Discussion

The rapid development of single-cell transcriptomic methods in recent years has greatly enhanced our understanding of GF biology, uncovering the heterogeneity of stromal cell populations in the oral mucosa and emphasizing the important role of GFs in driving inflammatory processes in periodontitis^15,20^. Despite growing insights into the mechanisms and consequences of GF interaction with other cell types in gingival tissue, such as neutrophils^15,16^, the controversy remains regarding their influence on osteoclast formation and, consequently, alveolar bone resorption. While studies of healthy GFs cultured under homeostatic conditions have consistently reported an inhibitory effect of their secreted products on RANKL-induced osteoclastogenesis^17,18^, analyses of GFs exposed to bacterial infection or stimulation with bacterial components have yielded conflicting results. Some studies reported suppression of RANKL-mediated osteoclast formation by conditioned media from GFs infected with *P. gingivalis* or stimulated with *Escherichia coli* LPS^17,18^, whereas others suggested a pro-osteoclastogenic role upon infection with *P. gingivalis* or treatment with subgingival biofilms, attributed to a shift in the RANKL/OPG expression ratio favoring osteoclastogenesis^19,21^. In the present study, we demonstrate that, irrespective of the absence or presence of bacterial infection or cytokine stimulation in vitro, conditioned media derived from healthy donor GFs significantly inhibit RANKL-induced osteoclast formation and bone resorption. This inhibitory effect is associated with the production of high levels of OPG, which neutralize exogenous RANKL. Importantly, we did not detect RANKL production by GFs cultured either in 2D monocultures or in RHG organotypic cultures. This is consistent with previous findings^17^ and, to the best of our knowledge, biologically relevant levels of RANKL protein have never been reported in GF monocultures. These observations suggest that, despite detectable RANKL mRNA expression and its modulation by bacterial factors, GFs are not a significant source of RANKL in gingival tissue.

Some reports describe the possibility of osteoclastogenesis induction by conditioned media from GFs in the absence of exogenous RANKL^22,23^. It has been speculated that this process is driven by pro-inflammatory cytokines such as TNF or IL-6 and could explain the formation of osteoclast-like cells even in the presence of high OPG levels^24^. However, the concept of RANKL-independent osteoclastogenesis remains controversial, and several researchers question the existence of such alternative pathways^25,26^. In light of these controversies, recent attention has shifted to MNGCs. These cells originate from monocytes or macrophages and share both morphological and molecular features with osteoclasts, including the expression of certain osteoclast-associated markers such as TRAP, but lack the ability to resorb bone^27^. Notably, MNGC formation is triggered by pro-inflammatory cytokines and occurs independently of the OPG/RANKL axis^27,28^. Since the bone-resorbing activity of osteoclast-like cells induced by GF-derived conditioned media in the absence of RANKL has not been assessed^22,23^, the possibility that the observed cells are MNGCs rather than functional osteoclasts cannot be ruled out. Accordingly, future studies on GF-mediated regulation of osteoclastogenesis should include a detailed functional characterization of the observed cell populations.

Here, we demonstrate for the first time that the production of OPG is significantly decreased in GFs from patients with periodontal disease, which correlates with their reduced ability to suppress osteoclast formation compared to cells from healthy individuals. It is well established that GFs isolated from inflamed gingival tissue of periodontitis patients retain some inflammatory properties such as increased expression of pattern recognition receptors, as well as elevated production of inflammatory cytokines, chemokines, and reactive oxygen species^29–31^. These differences are further amplified upon GF stimulation with LPS or infection with *P. gingivalis*^29,32^. Consistently, we found that the most notable difference in OPG production between GFs from healthy donors and periodontitis patients occurs after TNF stimulation and *P. gingivalis* infection. These findings suggest that prolonged exposure to the inflammatory microenvironment or to oral pathogens could induce a form of transcriptional memory in GFs that persists in vitro. Although the concept of trained immunity was originally described in innate immune cells, emerging evidence indicates that non-immune cells, such as fibroblasts, can also undergo long-lasting functional reprogramming^33^. Indeed, a recent study showed that LPS from *P. gingivalis* enhanced GF activation in response to a second LPS challenge one week after the initial stimulation^34^.

While further studies are necessary to understand the mechanism(s) underlying diminished OPG production by GFs from patients with periodontitis, it is likely that epigenetic regulatory mechanisms are involved. These mechanisms, which include DNA methylation and posttranslational histone modifications^35^, have been linked to OPG regulation in many diseases. Methylation of regulatory regions of the *OPG* gene is associated with reduced gene expression^36^ and hypermethylation of the *OPG* promoter has been reported in femoral bone tissues of patients with osteoporosis^37^, highlighting its critical role in maintaining bone homeostasis. Interestingly, OPG deficiency caused by high levels of promoter methylation has also been observed in several cancer cell lines^38,39^. The persistent changes in OPG expression may also result from changes in the acetylation level of N-terminal lysine residues on histone proteins, catalyzed by histone acetyltransferases (HATs) and histone deacetylases (HDACs). Although the direct impact of HATs or HDACs on *OPG* expression in GFs has not yet been investigated, studies in mice have shown that Hdac3 deletion in skeletal progenitor cells increases *Opg* expression in calvarial bone tissue and elevates Opg serum levels^40^. Notably, HDAC3 activity is also essential for the transcriptional induction of several inflammatory mediators associated with periodontitis in GFs^41^. These findings underscore the potential importance of epigenetic mechanisms in regulating the RANKL/OPG axis in GFs. Given the anti-inflammatory and bone-protective effects of HDAC inhibitors in preclinical models of periodontitis^35,42^, these observations also support investigation of the potential of targeting these mechanisms with epigenetic drugs to reverse the impairment of OPG production in periodontitis.

While the presented data indicate that OPG production is dysregulated in GFs from patients with periodontitis, our study has several limitations that should be addressed in future research. First, our cohorts of healthy individuals and periodontitis patients were not age-matched, with the healthy donor group being significantly younger. Although we did not observe any significant association between donor age and *OPG* mRNA expression (data not shown), previous studies in mouse fibroblasts have reported age-related differences in *Opg* mRNA levels^43^. Given the known differences in the biological properties of human GFs from young and elderly donors^44^, as well as established age-related changes in the epigenome^45^, future independent studies in age-matched cohorts are warranted. Second, we used pooled conditioned media from different GF donors in osteoclastogenesis and bone resorption assays. This approach preserves overall differences between the tested groups but does not account for inter-individual variability between cells from different donors. These analyses should therefore be expanded to include comparisons of individual GF lines and larger sample sizes. Third, the differences in OPG production by GFs from healthy donors and periodontitis patients observed in long-term in vitro cultures – which can influence many aspects of cellular phenotypes – should be independently confirmed through in situ analyses of intact gingival tissue samples or by single-cell RNA-seq datasets. Finally, our functional studies relied on siRNA-mediated gene silencing, an approach that does not result in complete elimination of the target protein. The residual OPG protein levels may have contributed to the incomplete blockade of the anti-osteoclastogenic effect of GFs observed in our experiments.

In summary, our findings suggest that GFs may exert differential effects on osteoclastogenesis depending on whether they operate under homeostatic or inflammatory conditions. While in health they have a bone-protective role by serving as the primary source of OPG in gingival tissue, in periodontitis they may indirectly promote bone resorption due to a reduced ability to produce OPG. These observations also highlight the limitations of relying on healthy donor-derived GFs, which can be easily obtained during third molar extraction procedures, in studies exploring pathogenic processes related to periodontitis. Interestingly, despite the common belief that inflammatory activation promotes osteoclastogenesis, we found that conditioned media from GFs exposed to acute TNF stimulation in vitro were even more effective at suppressing osteoclast differentiation. This effect may result from increased OPG production or the secretion of other anti-osteoclastogenic factors, such as PGE2^46^. Therefore, our results not only suggest a possible new pathogenic role of GFs in periodontitis but also provide evidence that these cells could undergo long-lasting changes that may not be fully reversible after periodontal treatment, opening an important new avenue for future research.

## Materials and methods

### Subjects, cell isolation, and culture

Gingival tissue specimens were obtained from healthy individuals undergoing the surgical phase of orthodontic treatment at the Chair of Oral Surgery or from patients with periodontitis at the Department of Periodontology, Preventive Dentistry and Oral Medicine (both at the Faculty of Medicine, Jagiellonian University, Krakow, Poland). Sample collection was approved by the Bioethical Committee of the Jagiellonian University (opinion numbers 1072.6120.104.2019 and 1072.6120.74.2023) and all methods were performed in accordance with the regulations and guidelines of the Committee. Participants included in the study were non-smokers, non-pregnant, and free from systemic diseases, recent antibiotic therapy, or a history of chemo-/radiotherapy. All participants provided written informed consent prior to participation in the study. Their demographic and clinical characteristics are presented in Supplementary Table S1. GFs were isolated and cultured as previously described^47^. One day prior to experiments, GFs were seeded in antibiotic-free DMEM (VWR) supplemented with 2% FBS (Sigma Aldrich).

Peripheral blood from deidentified healthy donors was purchased from the Regional Blood Donation and Transfusion Center (Krakow, Poland). Monocytes were isolated using Pancoll (Pan Biotech) density-gradient centrifugation, followed by magnetic-activated cell sorting (Miltenyi Biotec) as described before^48^. Cells were seeded in 24 well plates (500,000 cells/well) in αMEM (Gibco) containing 10% FBS and M-CSF (25 ng/ml, BioLegend) and cultured for 2 days. After this initial period, the medium was replaced with αMEM containing 10% FBS, either alone or mixed in a 1:1 ratio with GF-derived conditioned medium (also prepared in αMEM with 10% FBS). All culture conditions were further supplemented with M-CSF (10 ng/ml) with or without RANKL (10 or 40 ng/ml). The medium, including supplements, was refreshed twice weekly, and cultures were maintained for 19 days to allow for osteoclast differentiation.

### Bacterial culture and cell infection

*P. gingivalis* ATCC 33,277 were grown anaerobically on blood agar plates supplemented with brain–heart infusion (Becton–Dickinson), yeast extract, L-cysteine (0.5 mg/ml), hemin (10 µg/ml) and vitamin K (0.5 µg/ml). After 3–4 days bacteria were inoculated into liquid culture and bacterial suspensions at optical density (OD)_600_=1 (equivalent to 10^9^ colony-forming units/ml) were prepared and used for cell infection, as described previously^41^.

### Construction and culture of reconstructed human gingiva (RHG)

RHG was constructed as described elsewhere^49^. Briefly, GFs from healthy donors were mixed with a collagen I solution (4 mg/ml; the collagen isolation protocol is detailed in Smola et al.^50^) and seeded onto 6-well transwell inserts with 0.4 mm pore membrane (Corning) (130,000 cells per insert). Alternatively, collagen I gels without GFs were applied to the inserts. After 24 h, telomerase-immortalized gingival keratinocytes (TIGKs) were seeded on top of the GF-populated matrix (500,000 cells per insert). Cells were incubated for 3 days in DMEM supplemented with 5% FBS, 20% HamsF12 (Gibco), 1% penicillin–streptomycin (Gibco), EGF (2 ng/ml) hydrocortisone (1 µM), insulin (100 nM) and isoproterenol (1 µM) (all from Sigma Aldrich). Subsequently, RHGs were raised to the air–liquid interface and maintained in DMEM supplemented with FBS (1%), HamsF12 (20%), EGF (2 ng/ml), l-ascorbic acid (130 µg/ml), penicillin–streptomycin (1%), hydrocortisone (1 µM), insulin (100 nM), isoproterenol (1 µM), carnitine (10 µM, Sigma-Aldrich) and L-serine (10 mM, Sigma-Aldrich). The culture medium was refreshed twice weekly, and RHGs were cultured for a total of 11 days. One day prior to TNF stimulation (1 ng/ml), the medium was replaced with DMEM containing all supplements except for hydrocortisone and antibiotics. After 24 h of stimulation, supernatants were collected for ELISA, a fragment of the RHG was excised for paraffin embedding, and GFs were separated from TIGKs for subsequent RNA isolation.

### Preparation of GF-derived conditioned media

GFs were seeded in 6-well plates (500,000 cells/well) in antibiotic-free DMEM containing 2% FBS. Cells were then infected with *P. gingivalis* (MOI = 20) in the presence or absence of TNF (10 ng/ml) for 24 h. Alternatively, GFs were transfected with control or *OPG*-targeting siRNA as described in the online Supplement, followed by stimulation with TNF (10 ng/ml) for 24 h. Subsequently, cells were washed, incubated in antibiotic-containing medium, washed again and subsequently cultured in αMEM supplemented with 10% FBS for 24 h. The detailed procedure was described previously^16^ and is schematically shown in Fig. 1A. Conditioned media from different healthy donors or patients with periodontitis were pooled prior to experiments.

### TRAP staining and osteoclast area assessment

Osteoclasts were fixed with 4% paraformaldehyde (Thermo Scientific) for 10 min at RT. Cells were then stained for one minute at RT with Fixative Solution (37% formaldehyde, acetone, Citrate Solution). This was followed by TRAP staining using a Leukocyte acid phosphatase kit (Sigma-Aldrich) according to the manufacturer’s instructions. Nuclei were counterstained with DAPI (Thermo Scientific) for 5 min at RT. Stained cells were imaged using an Axio Observer 7 microscope (Zeiss), with five representative images taken per experimental condition. The average osteoclast area in each image was quantified using ImageJ software.

### Bone resorption assay

Bone resorption was analyzed using bovine cortical bone slices, kindly provided by Prof. Teun de Vries (ACTA, The Netherlands) as described before^51^. Briefly, bone slices were placed into 96-well plate. After that, monocytes were seeded at the density of 200,000 cells/well in αMEM containing 10% FBS and M-CSF (25 ng/ml). After 48 h the medium was replaced with αMEM containing 10% FBS, M-CSF (10 ng/ml), RANKL (10 ng/ml) and GFs-derived conditioned media (1:1 ratio). Cells were cultured for 19 days with medium changes performed twice weekly. Bone slices were then washed with distilled water, followed by incubation in 0.25 M NH4OH (Avantor) and sonication in ice water for 30 min. Subsequently, the bone slices were washed, incubated in KAl(SO4)2*12H2O for 10 min at RT and washed again. The resorption pits were visualized by staining the bone slices with a 1:1 mixture of Coomassie Brilliant Blue (Serva) and 20% acetic acid (Avantor). Images were acquired using an Axio Observer 7 microscope, with five representative images captured per condition. The resorbed areas were quantified using ImageJ software.

### RNA isolation and quantitative PCR (qPCR)

GFs were seeded in 12-well plate (250,000 cells/well), infected with *P. gingivalis* (MOI = 20) in presence or absence of TNF (10 ng/ml), and incubated for 4 h. Alternatively, RHG was prepared as described in the Materials and Methods section. GFs fraction were incubated in RPMI 1640 (Gibco) containing DNase I (0.1 mg/ml, Roche), collagenase P (0.2 mg/ml, Sigma Aldrich) and dispase (0.8 mg/ml, Gibco) at 37 °C for 15 min. TIGKs were incubated in trypsin-EDTA (BioWest) for 15 min in 37 °C. Total RNA was extracted using GeneMATRIX kit (Eurex) and quantified with a BioPhotometer D30 (Eppendorf). RNA were converted to cDNA using High-Capacity cDNA Reverse Transcription Kit (Applied Biosystems). Sequences of the primers (purchased from Merck) used in this study are listed in Table 1. qPCRs were carried out on CFX96 Touch Real-Time PCR Detection System (Bio-Rad), using PowerUp SYBR Green PCR mix (Applied Biosystems). Data were analyzed using the CFX Manager software (Bio-Rad). mRNA expression was calculated relative to *RPLP0* (for GFs in 2D monocultures), or to the mean of *RPLP0* and *GAPDH* expression (for GFs and TIGKs extracted from RHG).

**Table 1:** Sequences of primers used for qPCR analyses.

Gene	Forward primer	Reverse primer
*RPLP0*	GCGTCCTCGTGGAAGTGACATCG	TCAGGGATTGCCACGCAGGG
*GAPDH*	GCCAGCCGAGCCACATC	TGACCAGGCGCCCAATAC
*OPG*	CTGCGCGCTCGTGTTTC	ACAGCTGATGAGAGGTTTCTTCGT
*RANKL*	CGTTGGATCACAGCACATCAG	GTACCAAGAGGACAGACTCAC

### ELISA

GFs were seeded in 96-well plates (25,000 cells/well) and infected with *P. gingivalis* (MOI = 20) in the presence or absence of TNF (10 ng/ml). After 24 h, cell-free supernatants were collected for analysis. Osteoclasts were cultured as described above, and on day 19 of culture, supernatants were collected for analysis. Alternatively, recombinant human RANKL (10 ng/ml) was incubated with varying concentrations of recombinant human OPG (1, 5, and 10 ng/ml; R&D Systems) in PBS supplemented with 1% bovine serum albumin for one hour. The concentrations of OPG or RANKL were determined using OPG and RANKL Duo Sets (R&D Systems), respectively, according to the manufacturer’s instructions. Absorbance was measured using a FleXStation3 Multi-Mode Reader (Molecular Devices).

### Hematoxylin and eosin (H&E) staining

A 1–2 mm strip of RHG was fixed in 4% paraformaldehyde for 24 h and subsequently embedded in paraffin (Thermo Fisher). Tissue Sect. (5 μm) were prepared and stained with hematoxylin and eosin (H&E) using the following protocol: two washes in xylene (Avantor) (10 min each), two washes in 100% ethanol (6 min each), followed by 96% ethanol (5 min), 70% ethanol (5 min), and distilled water (5 min). Sections were then stained with hematoxylin (Sigma-Aldrich) (3 min), rinsed in running tap water (30 s), counterstained with eosin (Sigma Aldrich) (5 s), and dehydrated through 96% ethanol (6 min × 2) and 100% ethanol (10 min × 2). Finally, slides were cleared in xylene (15 min). After staining, the sections were dehydrated, air-dried, and mounted with coverslips using a xylene-based mounting medium (Epredia). The images were taken using a Nikon Eclipse 80i microscope at 100x magnification.

### Transfection with siRNA

GFs were seeded on 6-well plates (250,000 cells/well) in antibiotic-free DMEM containing 10% FBS. After 24 h, the culture medium was replaced with Opti-MEM Reduced Serum Medium (Thermo Scientific). OPG-targeting siRNA and non-targeting control siRNA (both from Dharmacon) were prepared at a final concentration of 20 nM by mixing with DharmaFECT1 (Dharmacon) and Opti-MEM, followed by incubation at RT for 20 min. The transfection mixtures were then added to the cells, which were incubated for 24 h. After that, the medium was replaced with DMEM containing 10% FBS, and cells were cultured for an additional 24 h. Following this incubation, the medium was again replaced with DMEM supplemented with 2% FBS and used for experiments.

### Single cell RNA-seq data preprocessing and analysis

Raw data was obtained from the GEO Accession Viewer (GSE164241: healthy gingival tissue samples)^15^. Sample-specific AnnData objects were generated and subsequently merged into a single dataset. Quality-control filtering was applied to remove low-quality cells and genes according to the following thresholds: cells with < 200 or >6000 detected genes, genes expressed in < 3 cells, cells with >15% mitochondrial gene expression, and outlier cells identified by a median absolute deviation ≥ 5. Scrublet was used to detect and exclude doublets. Raw counts were normalized and log-transformed, and highly variable genes were identified using Scanpy’s default parameters. Gene names were unified across samples using GENECODE v49 annotation. Dimensionality reduction was performed using PCA (number of principal components = 50), followed by construction of k-nearest-neighbor graph and Leiden clustering (resolution = 0.05). Cell types were annotated based on established marker genes reported in the original study including endothelial, fibroblast, immune and epithelial cells. UMAPs and dot-plots were generated, including visualization of the gene of interest *TNFRSF11B*, which encodes OPG.

### Statistical analysis

Data are presented as mean + SEM or + SD, where appropriate. The values of “n” refer to independent experiments performed on GFs or monocytes from different donors unless indicated otherwise. Comparisons between groups were performed using ordinary one-way ANOVA followed by Bonferroni multiple comparison test, unpaired t-test, or paired t-test, where appropriate. P values < 0.05 were considered statistically significant.

## Supplementary Information

Below is the link to the electronic supplementary material.

Supplementary Material 1

## Data Availability

The original contributions presented in the study are included in the article and supplemental material. Raw data are available from the corresponding author upon request. The single-cell RNA-seq dataset analyzed in this study was originally described by Williams et al.^15^ and is available in the GEO database under accession number GSE164241.
