# Supplementary material for: Diminished osteoprotegerin production by gingival fibroblasts from periodontitis patients is associated with their reduced ability to suppress osteoclastogenesis
Source: Sci Rep. 2025 Nov 28;16:449. doi: 10.1038/s41598-025-29944-w (PMC12774911; doi:10.1038/s41598-025-29944-w)
Supplement: Supplementary file 1 — Supplementary Material 1 [file 41598_2025_29944_MOESM1_ESM.pdf]

Supporting information

Table S1. Clinical and demographic characteristics of healthy donors (HD, n = 9) and periodontitis patients (n = 8) included in the study.

Characteristic	HD	periodontitis
Age (y)	33 (19-66)	57.5 (46-70)
Male:female ratio (n)	4:5	4:4
API [%]	n/a	30 (10-60)
SBI [%]	n/a	27.5 (5-56)
PPD [mm]	n/a	9 (7-11)
CAL [mm]	n/a	10 (9-12)

The values are presented as median (range), unless otherwise indicated. API: approximal plaque index; SBI: sulcus bleeding index; PPD: probing pocket depth; CAL: clinical attachment loss; n/a: not available.
